# What to Observe at the Bed-Side and after Death in Medical Cases

**Published:** 1855-04

**Authors:** 


Art. VIII.—*What to Observe at the Bed-side and after Death in Medical Cases.* Published under the authority of the London Medical Society of Observation. Second American, from the second and enlarged London edition. Philadelphia. Blanchard & Lea. 1855. 8vo. pp. 228.

Not more than a twelve-month ago we noticed at some length in this journal the little volume which bears the above title. We took occasion in that notice, after having indicated the objects of the work, to recommend it in the strongest terms to our readers and especially to those of them who are also our correspondents. The adoption of a uniform plan of arranging records would greatly lessen the labor of analyzing and comparing clinical observations, would rob the task of reporting cases of half its terrors, in fact would render it easy, and add immensely to their value and interest. "The Society believes that this scheme or method of arranging the clinical and anatomical phenomena of disease, will prove useful both to those who desire to learn systematically with what amount of detail, and in what order, those phenomena should be looked for, and also to those who wish to record with accuracy the results of their experience. It will probably be admitted, too, that errors in diagnosis are more frequently traceable to forgetfulness in searching for all possible evidences of disease, than to misinterpretation of those actually discovered."

